# Awareness and attitudes of parents towards pediatric dentistry and children's oral health: a cross-sectional study

**DOI:** 10.3389/froh.2026.1876757

**Published:** 2026-06-30

**Authors:** Ajay H. T. Rao, Khushi D. Shetty, Sundeep K. Hegde, Sharan S. Sargod

**Affiliations:** Department of Pediatric and Preventive Dentistry, Yenepoya Dental College, Yenepoya (Deemed to be University), Mangalore, India

**Keywords:** children's oral health, cross-sectional study, parental attitudes, parental awareness, pediatric dentistry, preventive dental care, socioeconomic factors

## Abstract

**Introduction:**

Parents play a pivotal role in shaping children's oral health behaviors and practices. This study aimed to assess parental awareness and attitudes regarding children's oral health and pediatric dental care, and to evaluate the influence of socioeconomic factors on these parameters.

**Materials and methods:**

A cross-sectional questionnaire-based study was conducted from June to December 2025 among parents of children aged below 10 years. Participants were recruited using convenience sampling from the Departments of Pediatrics and Pediatric and Preventive Dentistry at Yenepoya University. Data were collected using a Google Forms-based questionnaire. The sample size was calculated using Cochran's formula, yielding a final sample of 384 participants. Data were analyzed using the Pearson's chi-square test and multivariable binary logistic regression, with simultaneous adjustment for education and income. Statistical significance was set at *p* < 0.05.

**Results:**

Regarding parental awareness, 53.6% acknowledged that problems in deciduous teeth can affect permanent dentition, 89.1% knew the recommended brushing frequency, 85.4% recognised the importance of diet in oral health, and 91.7% acknowledged pediatric dentistry as a dental specialty. However, 46.1% were uncertain about the benefits of fluoride, and only 33.9% identified all recommended preventive measures against dental caries. Regarding parental attitudes, 70.1% believed that the first dental visit should be delayed until a problem arose, while 67.7% believed deciduous teeth deserved equal care as permanent teeth. Moreover, 90.6% of parents sought help from a pediatric dentist. Education and income significantly influenced parental awareness and attitudes on multiple parameters (*p* < 0.05). Multivariable logistic regression (adjusted for both education and income simultaneously) revealed that higher education was the strongest independent predictor of fluoride awareness (adjusted OR = 3.46, 95% CI: 2.18–5.50, *p* < 0.001), while neither education nor income independently predicted appropriate first dental visit timing (*p* > 0.05 for both).

**Conclusions:**

Parental awareness regarding basic oral hygiene practices was comparatively high; however, knowledge regarding fluoride, preventive measures, and the significance of primary dentition was relatively low. Socioeconomic status is a crucial independent determinant of parental awareness and attitude. The universal attitude of delayed first dental visits warrants population-wide health education campaigns.

## Introduction

Oral health is a crucial element of general health and wellness (1, 2). Early childhood is a crucial phase when lifelong habits regarding oral hygiene can be developed, in light of the fact that preventive actions taken at this point may have significant bearing on the oral health status achieved by the individual in their adulthood (3). Inadequate oral health among children may lead to problems like pain, inability to eat, speech issues, and low self-confidence levels (4).

Parents assume an essential role in building the health behavior patterns of their wards. In other words, they are key caregivers, role models, and decision-makers (5). The awareness, attitudes, and practices of parents directly impact the behavior of their children towards oral health practices and their usage of dental health care services (6).

Studies have revealed a strong link between parental knowledge and the oral health condition of children (7, 8).

Consequently, it is important that parents possess sufficient awareness and skills regarding dental health care (2).

Pediatric dentistry involves prevention and treatment of various disorders affecting oral health in infants, children, and adolescents (9).

The success of such preventive measures largely depends on involvement of parents because parents initiate and maintain proper oral health practices in infants. However, although community- and school-based oral health campaigns seek to make people more aware of children's oral health issues, such programs will not yield any success without sufficient parental awareness and interest in preventive care for children's teeth (10).

Despite growing awareness about the need for parental participation in children's oral health, certain gaps in awareness among parents still exist among different socioeconomic groups. Therefore, understanding these gaps can help develop effective interventions aimed at enhancing oral health in children.

In line with the above discussion, the aim of the current cross-sectional study was to evaluate parental awareness and attitudes regarding pediatric dentistry and their children's oral health and to evaluate the influence of socioeconomic factors on these outcomes.

## Materials and methods

### Study design and survey method

A cross-sectional questionnaire-based study was conducted between June and December 2025. Parents attending the Outpatient Departments of Pediatrics and Pediatric and Preventive Dentistry at Yenepoya University for their first visit during the study period were approached and invited to participate using a convenience sampling method. Following informed consent, eligible participants completed a Google Forms-based electronic questionnaire in the clinic using their personal electronic devices. The structured questionnaire comprised 16 questions covering sociodemographic variables, parental awareness, and attitudes towards children's oral health and pediatric dentistry. The questionnaire was developed following a comprehensive review of the literature and was adapted from previously published questionnaires assessing parental awareness, attitudes, and practices related to children's oral health and pediatric dentistry (9, 11–14). Items were modified and contextualised to align with the objectives of the present study and the local study population. Questionnaire content validity was assessed by an expert panel comprising one internal and two external subject experts; modifications were made based on their feedback to improve clarity and relevance. A pilot study was conducted among 30 parents to assess clarity, comprehensibility, and feasibility. Minor modifications were made based on participant feedback, and pilot participants were excluded from the final study sample.

Prior to participation, all respondents provided informed consent. Participation was voluntary, and each respondent could complete the questionnaire only once to prevent duplicate responses. No personally identifiable data were collected to ensure anonymity.

### Questionnaire validation

#### Content validity index (CVI) and average congruency percentage (ACP)

Content validity was assessed by a panel of three expert reviewers. Each of the 16 items was rated on a four-point relevance scale. The Item-level Content Validity Index (I-CVI), Scale-level CVI (S-CVI/UA and S-CVI/Ave), and Average Congruency Percentage (ACP) were computed. All 16 items achieved an I-CVI of 1.00 (unanimous expert agreement). Two items required minor wording clarification after the initial review round. Following revision, the S-CVI/Ave was 1.000, S-CVI/UA was 1.000, and the ACP was 100%, all exceeding recommended benchmarks (33), indicating excellent content validity.

### Inter-rater reliability (Cohen's kappa)

To assess inter-rater reliability in response coding, two trained data collectors independently categorised the responses of 30 pilot participants. Inter-rater reliability testing was conducted because, although the questionnaire consisted primarily of fixed-response items, categorisation of some items required interpretive judgment by data collectors (e.g., classifying ambiguous or partially completed responses, and coding multi-option responses into predefined categories). Assessing agreement between two independent coders prior to full-scale data entry was therefore considered essential to ensure consistency and reproducibility of the data coding process. Cohen's kappa (*κ*) was computed for each item. Item-level kappa values ranged from 0.651 to 1.000, with an overall mean *κ* of 0.831, indicating almost perfect inter-rater agreement (34). The per-item kappa values are presented in Table 1.

**Table 1:** Questionnaire validation: inter-rater reliability and test–retest reliability for all 16 items (pilot *n* = 30, 2-week interval).

No.	Item	Inter-rater Kappa (*κ*)	Interpretation (Inter-rater)	Test–retest Kappa (*κ*)	Interpretation (Test–retest)
Q01	Primary teeth affect permanent teeth	1.000	Almost perfect	0.783	Substantial
Q02	Education level (sociodemographic)	1.000	Almost perfect	1.000	Almost perfect
Q03	Income level (sociodemographic)	1.000	Almost perfect	1.000	Almost perfect
Q04	Fluoride toothpaste: safe and beneficial	0.927	Almost perfect	1.000	Almost perfect
Q05	Nighttime bottle-feeding causes decay	1.000	Almost perfect	0.651	Substantial
Q06	Oral habits linked to dental problems	1.000	Almost perfect	1.000	Almost perfect
Q07	All preventive measures identified	0.930	Almost perfect	0.930	Almost perfect
Q08	Appropriate age for first dental visit	1.000	Almost perfect	1.000	Almost perfect
Q09	Crowns/RCT possible on deciduous teeth	0.000*	—(OA: 96.7%)	0.000*	—(OA: 96.7%)
Q10	Paediatric dentistry is a specialty	1.000	Almost perfect	1.000	Almost perfect
Q11	Equal care: deciduous vs. permanent teeth	0.889	Almost perfect	0.889	Almost perfect
Q12	Diet essential for child’s oral health	1.000	Almost perfect	1.000	Almost perfect
Q13	Frequency of dental checkups	1.000	Almost perfect	1.000	Almost perfect
Q14	Type of dentist consulted for child	1.000	Almost perfect	1.000	Almost perfect
Q15	Tooth brushing frequency (twice daily)	1.000	Almost perfect	1.000	Almost perfect
Q16	Most important daily oral health habit	0.889	Almost perfect	0.889	Almost perfect
	Overall Mean *κ* (all 16 items)	0.915†	Almost perfect	0.884†	Almost perfect
	Mean *κ* (excluding Q09)	0.976	Almost perfect	0.943	Almost perfect
	ICC (overall score; 95% CI: 0.958–0.990)	—	—	0.980^‡^	Excellent

OA, observed agreement. Landis and Koch (34): *κ*: 0.61–0.80 = Substantial; *κ* > 0.80 = Almost perfect. Cicchetti (35): ICC ≥ 0.90 = Excellent.

* *κ* = 0.000 for Q09 is a mathematical artefact: both raters showed ≥96.7% agreement, but near-unanimous marginal distributions cause kappa to approach zero despite high observed agreement (Feinstein & Cicchetti, 1990).

† Mean *κ* computed across all 16 items (including Q09).

‡ ICC (two-way mixed model, absolute agreement) for total questionnaire score (sum of binary scores across 16 items, range: 0–16).

### Test–retest reliability

The temporal stability of the questionnaire was evaluated by administering it to a pilot subsample of 30 parents on two occasions separated by a two-week interval. The overall questionnaire score used for ICC computation was derived by summing the binary correct/incorrect scores across all 16 items (scored 0 or 1 per item), yielding a total score ranging from 0 to 16. The Intraclass Correlation Coefficient (ICC; two-way mixed model, absolute agreement) was computed for overall questionnaire scores. The ICC was 0.980 (95% CI: 0.958–0.990), indicating excellent test–retest reliability (Cicchetti (35): ≥0.90 = excellent). Per-item Cohen's kappa for knowledge-based items ranged from 0.651 (substantial) to 1.000 (almost perfect), confirming stable individual item responses over time. These pilot participants were not included in the final study sample. The pilot validation data (*n* = 30) are summarised in Table 1.

### Study subjects

The study was conducted among parents visiting the Outpatient Departments of Pediatrics and Pediatric and Preventive Dentistry at Yenepoya University during their first visit to either department. Convenience sampling was used for participant recruitment. Eligible participants were parents of children aged below 10 years working in non-dental professions, to minimise professional knowledge bias. Parents of children with special health care needs due to systemic or syndromic disorders were excluded.

It is acknowledged that recruiting participants from a healthcare setting may introduce selection bias, as these parents may possess higher health awareness than the general population. Participants were recruited from both the Department of Pediatrics and Pediatric and Preventive Dentistry. The survey was administered in-clinic (via the participants’ personal electronic devices).

### Sample size determination

The sample size was determined using the Cochran's formula, using a 95% confidence level, 5% margin of error, and a proportional parameter (*p*) of 0.5 to ensure maximum sample size estimation. Taking these parameters into consideration, the determined sample size was 384. Accordingly, 384 parents were recruited for this study.

### Variables assessed

The primary outcome variables were parental awareness and attitudes towards children's oral health and pediatric dentistry. Socioeconomic factors—specifically education level and monthly household income—were treated as independent variables.

### Statistical analysis

Data were entered in Microsoft Excel and analyzed using Jamovi software (version 2.3) and supplementary statistical computation. Categorical variables were reported as frequencies and percentages. The Pearson chi-square (*χ*^2^) test was used to assess associations between sociodemographic variables and awareness/attitude responses. Multivariable binary logistic regression was performed to estimate adjusted odds ratios (aOR) with 95% Confidence Intervals (95% CI) for key outcome variables. Separate binary logistic regression models were constructed for each dichotomised awareness and attitude outcome. Education (higher vs. non-higher education, reference: non-higher) and income (>₹25,000/month vs. ≤₹25,000/month, reference: ≤₹25,000/month) were entered simultaneously into each model to allow mutual adjustment. This approach allows each predictor's independent contribution to each outcome to be estimated after controlling for the other socioeconomic variable. Statistical significance was set at *p* < 0.05. Questionnaire reliability was assessed using the Intraclass Correlation Coefficient (ICC) for test–retest reliability and Cohen's kappa for both inter-rater and per-item test–retest agreement on a pilot subsample of 30 participants.

### Ethics statement

Ethical approval for the study was obtained from the Yenepoya Ethics Committee-2 prior to the commencement of the study (Approval No.: YEC2/2025/183). The study adhered to the ethical standards of the Declaration of Helsinki and to STROBE reporting guidelines. Participation was voluntary, and informed consent was secured from all participants before inclusion in the study. Participant anonymity and confidentiality were ensured, and no identifiable personal data was collected.

## Results

The study results are presented in Tables 2–5. Participant flow is illustrated in Figure 1 (see Supplementary Material).

**Table 2:** Sociodemographic characteristics and overall parental awareness and attitudes (*N* = 384).

Variable/Response	*n*	%
Education level		
Higher education	130	33.9
Middle school education	88	22.9
Secondary education	160	41.7
Primary education	6	1.6
Income level (₹/month)		
₹12,000–25,000	148	38.5
₹25,000–50,000	146	38.0
<₹12,000	52	13.5
>₹50,000	38	9.9
Age of parents		
<30 years	80	20.8%
30–45 years	292	76%
>45 years	12	3.1%
Gender		
Male	59	15.4%
Female	325	84.6%
Residence		
Urban	153	39.8%
Rural	231	60.2%
Number of children		
1	27	7%
2	95	24.7%
≥3	262	68.2%
Child age distribution		
<3 years	127	33.1%
3–5 years	172	44.8%
6–10 years	85	22.1%
Primary teeth problems affect permanent teeth?		
Yes	206	53.6
No	143	37.2
Don't know	35	9.1
Most important daily habit for child's oral health?		
Brushing teeth	185	48.2
Limiting sweets	180	46.9
Rinsing with water only	12	3.1
Don't know	7	1.8
Recommended tooth brushing frequency?		
Twice daily	342	89.1
Once daily	17	4.4
Three times or more	20	5.2
Don't know	5	1.3
Is fluoride in toothpaste safe and beneficial?		
Yes, safe and beneficial	151	39.3
Uncertain/Don't know	177	46.1
Unsafe	41	10.7
Has no effect	15	3.9
Nighttime bottle feeding causes tooth decay?		
Yes	263	68.5
No	121	31.5
Oral habits (thumb sucking/mouth breathing) linked to dental problems?		
Yes	311	81.0
No	44	11.5
Don't know	29	7.6
How to prevent dental caries in children?		
All of the above (correct)	130	33.9
Limit sugary snacks only	95	24.7
Supervise brushing only	43	11.2
Visit dentist regularly only	116	30.2
Child's first dental visit: at what age?		
By 6 months of age	46	12.0
Before first birthday	69	18.0
Only when a problem arises	269	70.1
Crowns/RCT possible on deciduous teeth?		
Yes	336	87.5
No	48	12.5
Aware that pediatric dentistry is a specialty?		
Yes	352	91.7
No	32	8.3
Do primary (deciduous) teeth need as much care as permanent teeth?		
Yes	260	67.7
No	95	24.7
Not sure	29	7.6
Is diet essential for child's oral health?		
Yes	328	85.4
No	31	8.1
Not sure	25	6.5
How often do you attend dental checkups?		
Once in 3 months	25	6.5
Once in 6 months	88	22.9
Once a year	86	22.4
Only when a problem arises	185	48.2
Which dentist do you consult for child's dental problems?		
Pediatric dentist	348	90.6
General dental practitioner	11	2.9
Pediatrician	25	6.5

**Table 3:** Comparison of education level with parental awareness and attitude responses across all 14 questions (*N* = 384).

Variable (Response coded)	Higher education (*n* = 130) *n* (%)	Middle education (*n* = 88) *n* (%)	Secondary education (*n* = 160) *n* (%)	Primary education (*n* = 6) *n* (%)	*χ*^2^ (df = 3)	*p*-value
Primary teeth affect permanent teeth (Yes)	88 (67.7)	24 (27.3)	93 (58.1)	1 (16.7)	39.5	<0.001***
Most important daily habit: Brushing selected	96 (73.8)	20 (22.7)	69 (43.1)	0 (0.0)	64.4	<0.001***
Brushing twice daily (Correct)	118 (90.8)	78 (88.6)	143 (89.4)	3 (50.0)	9.8	0.020*
Fluoride: safe and beneficial (Yes)	80 (61.5)	19 (21.6)	52 (32.5)	0 (0.0)	45.5	<0.001***
Nighttime bottle feeding hazard (Yes)	99 (76.2)	39 (44.3)	123 (76.9)	2 (33.3)	36.0	<0.001***
Oral habits linked to dental problems (Yes)	112 (86.2)	67 (76.1)	130 (81.2)	2 (33.3)	12.5	0.006**
All preventive measures identified (Yes)	63 (48.5)	7 (8.0)	59 (36.9)	1 (16.7)	40.2	<0.001***
Appropriate first dental visit (<1 yr)	42 (32.3)	19 (21.6)	53 (33.1)	1 (16.7)	4.5	0.208 NS
RCT/Crowns possible on deciduous teeth (Yes)	116 (89.2)	72 (81.8)	144 (90.0)	4 (66.7)	6.2	0.100 NS
Paediatric dentistry is a specialty (Yes)	117 (90.0)	82 (93.2)	148 (92.5)	5 (83.3)	1.4	0.699 NS
Primary teeth need equal care (Yes)	98 (75.4)	45 (51.1)	112 (70.0)	5 (83.3)	15.6	0.001**
Diet essential for oral health (Yes)	114 (87.7)	74 (84.1)	136 (85.0)	4 (66.7)	2.4	0.497 NS
Regular dental checkups (vs. only problem)	88 (67.7)	25 (28.4)	85 (53.1)	1 (16.7)	35.5	<0.001***
Consult paediatric dentist (Yes)	115 (88.5)	84 (95.5)	144 (90.0)	5 (83.3)	3.6	0.310 NS

NS, not significant; *χ*^2^, Pearson Chi-square statistic; df, degrees of freedom. All reported responses represent the “correct” or clinically appropriate response for awareness questions, or the “Yes” response for attitude questions.

* *p* < 0.05.

** *p* < 0.01.

*** *p* < 0.001.

**Table 4:** Comparison of income level with parental awareness and attitude responses across all 14 questions (*N* = 384).

Variable (Response coded)	₹25k–50k (*n* = 146) *n* (%)	₹12k–25k (*n* = 148) *n* (%)	<₹12k (*n* = 52) *n* (%)	>₹50k (*n* = 38) *n* (%)	*χ*^2^ (df = 3)	*p*-value
Primary teeth affect permanent teeth (Yes)	95 (65.1)	65 (43.9)	20 (38.5)	26 (68.4)	21.4	<0.001***
Most important daily habit: Brushing selected	69 (47.3)	62 (41.9)	30 (57.7)	24 (63.2)	7.7	0.053 NS
Brushing twice daily (Correct)	138 (94.5)	131 (88.5)	40 (76.9)	33 (86.8)	12.6	0.006**
Fluoride: safe and beneficial (Yes)	72 (49.3)	41 (27.7)	16 (30.8)	22 (57.9)	21.6	<0.001***
Nighttime bottle feeding hazard (Yes)	114 (78.1)	90 (60.8)	35 (67.3)	24 (63.2)	10.8	0.013*
Oral habits linked to dental problems (Yes)	125 (85.6)	123 (83.1)	31 (59.6)	32 (84.2)	18.1	<0.001***
All preventive measures identified (Yes)	57 (39.0)	39 (26.4)	16 (30.8)	18 (47.4)	8.8	0.032*
Appropriate first dental visit (<1 yr)	39 (26.7)	46 (31.1)	21 (40.4)	9 (23.7)	4.2	0.238 NS
RCT/Crowns possible on deciduous teeth (Yes)	136 (93.2)	127 (85.8)	39 (75.0)	34 (89.5)	12.2	0.007**
Paediatric dentistry is a specialty (Yes)	138 (94.5)	140 (94.6)	39 (75.0)	35 (92.1)	22.1	<0.001***
Primary teeth need equal care (Yes)	109 (74.7)	91 (61.5)	28 (53.8)	32 (84.2)	15.1	0.002**
Diet essential for oral health (Yes)	130 (89.0)	131 (88.5)	33 (63.5)	34 (89.5)	23.3	<0.001***
Regular dental checkups (vs. only problem)	88 (60.3)	58 (39.2)	28 (53.8)	25 (65.8)	16.7	<0.001***
Consult paediatric dentist (Yes)	136 (93.2)	138 (93.2)	39 (75.0)	35 (92.1)	17.3	<0.001***

NS, not significant; *χ*^2^, Pearson Chi-square statistic; df, degrees of freedom. Income in Indian Rupees (₹) per month. All reported responses represent the “correct” or clinically appropriate response for awareness questions, or the “Yes” response for attitude questions.

* *p* < 0.05.

** *p* < 0.01.

*** *p* < 0.001.

**Table 5:** Multivariable binary logistic regression—adjusted odds ratios for key outcome variables (education and income mutually adjusted, *N* = 384).

Outcome Variable	aOR (Education)	95% CI (Education)	*p* (Education)	aOR (Income)	95% CI (Income)	*p* (Income)
Primary teeth affect permanent teeth	1.94	1.22–3.08	0.005**	2.21	1.43–3.39	<0.001***
Fluoride: safe and beneficial	3.46	2.18–5.50	<0.001***	1.95	1.24–3.05	0.004**
All preventive measures identified	2.36	1.49–3.74	<0.001***	1.44	0.92–2.26	0.115 NS
Nighttime bottle feeding hazard	1.52	0.92–2.50	0.102 NS	1.61	1.02–2.55	0.041*
Primary teeth need equal care	1.41	0.86–2.31	0.179 NS	2.04	1.29–3.24	0.002**
Regular dental checkups	2.32	1.46–3.67	<0.001***	1.71	1.11–2.62	0.014*
Appropriate first dental visit (<1 yr)	1.36	0.84–2.21	0.210 NS	0.64	0.40–1.02	0.061 NS

aOR = Adjusted Odds Ratio (mutually adjusted for education and income in each model). 95% CI = 95% Confidence Interval. Education reference: non-higher education (*n* = 254). Income reference: ≤₹25,000/month (*n* = 200). NS, not significant.

* *p* < 0.05.

** *p* < 0.01.

*** *p* < 0.001.

### Participant characteristics

Among the 384 participants, the majority had secondary education (41.7%), followed by higher education (33.9%), middle school education (22.9%), and primary education (1.6%). Most participants fell within the monthly income brackets of ₹12,000–25,000 (38.5%) and ₹25,000–50,000 (38.0%), while 13.5% earned less than ₹12,000 and 9.9% earned more than ₹50,000 per month. Most participants were aged 30–45 years (76.0%). Most respondents were mothers (84.6%), 60.2% resided in rural areas, and 68.2% had three or more children. The study population consisted of parents attending the Department of Pediatrics and the Department of Pediatric and Preventive Dentistry at Yenepoya University for the first time.

### Parental awareness

Regarding primary dentition, 53.6% of parents acknowledged that problems in deciduous teeth can impact permanent dentition, while 37.2% disagreed and 9.1% were unsure. Awareness was significantly associated with both education (*χ*^2^ = 39.5, *df* = 3, *p* < 0.001) and income (*χ*^2^ = 21.4, *df* = 3, *p* < 0.001), with higher levels among more educated and higher-income parents.

Concerning daily oral hygiene, 48.2% identified toothbrushing and 46.9% identified limiting sweets as the most important habit; 89.1% correctly knew the recommended brushing frequency (twice daily).

Fluoride awareness was notably low, with 46.1% uncertain about the safety and benefits of fluoridated toothpaste, and only 39.3% correctly identifying fluoride as safe and beneficial. Significant associations were found with education (*χ*^2^ = 45.5, *df* = 3, *p* < 0.001) and income (*χ*^2^ = 21.6, *df* = 3, *p* < 0.001). Among parents with higher education, 61.5% correctly identified fluoride as beneficial, compared to only 21.6% among those with middle school education.

Regarding risk factor awareness, 68.5% recognised the harmful effects of nighttime bottle feeding on dental health (*χ*^2^ = 36.0, *df* = 3, *p* < 0.001 for education; *χ*^2^ = 10.8, *df* = 3, *p* = 0.013 for income), and 81.0% acknowledged the link between harmful oral habits (thumb sucking, mouth breathing) and dental problems.

For preventive measures, only 33.9% of parents were able to identify all three recommended strategies (limiting sugary snacks, supervised brushing, and regular dental visits), with significant associations with education (*χ*^2^ = 40.2, *df* = 3, *p* < 0.001) and income (*χ*^2^ = 8.8, *df* = 3, *p* = 0.032).

### Parental attitudes

Regarding the first dental visit, 70.1% of parents believed it should occur only when a dental problem arises, while 12.0% recommended 6 months of age and 18.0% before the first birthday. This attitude was not significantly associated with education (*χ*^2^ = 4.5, *df* = 3, *p* = 0.208) or income (*χ*^2^ = 4.2, *df* = 3, *p* = 0.238).

Awareness of treatment options was high, with 87.5% recognising that procedures such as crowns and root canal treatment can be performed on deciduous teeth. Pediatric dentistry was recognised as a specialty by 91.7% of parents.

Regarding attitudes towards primary teeth care, 67.7% believed deciduous teeth require the same care as permanent teeth. Association with education was significant (*χ*^2^ = 15.6, *df* = 3, *p* = 0.001) and with income (*χ*^2^ = 15.1, *df* = 3, *p* = 0.002). Furthermore, 85.4% recognised the importance of diet in oral health.

For dental care-seeking behavior, 48.2% of participants reported visiting a dentist only when a problem arises (*χ*^2^ = 35.5, *df* = 3, *p* < 0.001 for education; *χ*^2^ = 16.7, *df* = 3, *p* < 0.001 for income). Regarding healthcare preference, 90.6% of parents reported consulting a pediatric dentist.

### Multivariable logistic regression analysis

Multivariable binary logistic regression with simultaneous adjustment for education and income revealed that higher education was the strongest independent predictor of correct fluoride awareness (aOR = 3.46, 95% CI: 2.18–5.50, *p* < 0.001) and of regular dental checkup behavior (aOR = 2.32, 95% CI: 1.46–3.67, *p* < 0.001), after adjusting for income. Higher income was independently associated with increased odds of awareness of primary teeth affecting permanent dentition (aOR = 2.21, 95% CI: 1.43–3.39, *p* < 0.001) and equal care for primary teeth (aOR = 2.04, 95% CI: 1.29–3.24, *p* = 0.002), after adjusting for education. Higher education independently predicted all preventive measures identification (aOR = 2.36, 95% CI: 1.49–3.74, *p* < 0.001), while income was not independently significant for this outcome after adjustment (aOR = 1.44, *p* = 0.115). Notably, neither education nor income independently predicted the likelihood of making an appropriate first dental visit (education aOR = 1.36, *p* = 0.210; income aOR = 0.64, *p* = 0.061), confirming that delayed first dental visits represent a universal attitude gap that transcends socioeconomic boundaries. Full adjusted regression results are presented in Table 5.

## Discussion

Oral health forms a key component of overall childhood health, impacting nutrition, development, and quality of life (15).The condition of primary teeth plays a key role in determining the future health status of permanent teeth (16).

Based on the theory of social cognitive learning, parental oral health awareness, attitude, and practices have a significant impact on children's health behaviors (17).

53.6% of parents in the present study were aware that there was an impact of problems occurring in primary teeth on permanent dentition. Despite indicating moderate awareness, the percentage is much lower compared to the proportions found by Doğan et al. (89.6%) and Wapniarska et al. (80.1%) but comparable to the proportions found in the study by Almalki et al. (68.7%) (9, 11, 18).

After adjusting for income, higher education remained a significant independent predictor of this awareness (aOR = 1.94, 95% CI: 1.22–3.08, *p* = 0.005), and after adjusting for education, higher income was also independently significant (aOR = 2.21, 95% CI: 1.43–3.39, *p* < 0.001), consistent with findings of Dogan et al. (*p* < 0.001) (19).

Primary teeth play an essential role in mastication, pronunciation, aesthetics, and guiding the permanent dentition in terms of eruption (19, 20). The findings from the current study indicate that 67.7% of parents were aware that primary teeth require equal care as permanent teeth.

This proportion is in line with the results reported by Patil et al. (72%) (21) but it deviates from studies with lower awareness, where parents underestimate the value of primary teeth since they eventually fall off (19, 22, 23). After multivariable adjustment, income was independently associated with this awareness (aOR = 2.04, 95% CI: 1.29–3.24, *p* = 0.002), while education was not independently significant (aOR = 1.41, *p* = 0.179) after controlling for income, suggesting that material resources may be a stronger determinant of this attitude than educational attainment *per se*.

In terms of oral hygiene habits, almost identical percentages of parents considered tooth-brushing (48.2%) and reduced sugar consumption (46.9%) as essential preventive factors. Moreover, 89.1% of the study subjects had knowledge of the correct brushing frequency, which should be done twice daily, and is comparable to the findings of Patil et al. (81.7%) (21).

Dietary knowledge was also high, with 85.4% aware of the impact of proper nutrition on dental health, which is consistent with prior research (24).

One notable finding of this study is the low level of awareness on fluoride. Around half of the subjects (46.1%) were not sure about the safety and advantages of using fluoridated toothpaste. This is consistent with earlier research on insufficient understanding of fluoride's function (2, 25).

After mutual adjustment, higher education was the strongest independent predictor of correct fluoride awareness (aOR = 3.46, 95% CI: 2.18–5.50, *p* < 0.001), representing the largest adjusted effect size observed. Higher income also remained independently significant (aOR = 1.95, 95% CI: 1.24–3.05, *p* = 0.004) after adjusting for education, indicating that both education and income independently contribute to fluoride knowledge. These findings underscore the need for dental professional education campaigns targeting lower-education populations (26, 27).This implies the need for better education on the part of dentists and other experts.

The awareness of risk factors like night-time feeding from bottles had a moderately high percentage of 68.5%, which was similar to the proportions observed in the study conducted by Fatani et al. (69.3%) (28). After multivariable adjustment, income was independently associated with bottle feeding awareness (aOR = 1.61, 95% CI: 1.02–2.55, *p* = 0.041), while education was not independently significant (aOR = 1.52, *p* = 0.102), suggesting that material resource differences may be the primary driver of this disparity.

In the same way, 81% of the parents understood the negative consequences of harmful oral behaviors, which is consistent with prior study by AlMugairin et al. (16).

Preventive behaviors varied, as only 33.9% of parents understood the significance of restricting sweets, monitoring tooth brushing, and visiting the dentist regularly. This suggests that there is still insufficient awareness of effective preventive measures, as noted in the study by Altamimi et al. (29). After adjustment, higher education remained independently significant (aOR = 2.36, 95% CI: 1.49–3.74, *p* < 0.001), while income was not independently significant (aOR = 1.44, *p* = 0.115), indicating that educational attainment is the primary independent determinant of comprehensive preventive knowledge.

Another problematic issue was that 70.1% of parents were of the opinion that the first dental appointment should be made only if there was a problem. However, this contradicts the guidelines set by the American Academy of Pediatric Dentistry and the American Dental Association that suggest the child's first dental visit should take place when he/she is a year old (30). However, there is evidence that this phenomenon is also widespread, highlighting that awareness remains insufficient (21, 23). Multivariable logistic regression revealed that neither education (aOR = 1.36, *p* = 0.210) nor income (aOR = 0.64, *p* = 0.061) independently predicted this attitude after mutual adjustment, confirming it as a universal knowledge gap that transcends socioeconomic boundaries. This finding has critical public health implications: it indicates that targeted messaging for low-income or low-education groups alone is insufficient—population-wide education campaigns are essential.

With regard to the frequency of dental visits, 48.2% of the respondents stated that they would visit a dentist only when problems arose, emphasizing a symptomatic approach to dental health care. Previous studies have shown that such behavior was more often observed in the case of individuals from poorer social strata (31, 32). After adjustment, both higher education (aOR = 2.32, 95% CI: 1.46–3.67, *p* < 0.001) and higher income (aOR = 1.71, 95% CI: 1.11–2.62, *p* = 0.014) were independently associated with regular dental checkups, suggesting that both education and income independently contribute to preventive dental care utilisation.

However, parents' awareness about pediatric dental therapy was relatively high, as 87.5% knew that root canals and crown therapy could be carried out on primary teeth. This is a higher rate compared to that observed in other studies where there was low awareness (21, 23). Moreover, majority of the parents were willing to seek help from a pediatric dentist (90.6%).

### Limitations

The present study has certain limitations that should be considered while interpreting the findings. Participants were recruited using a convenience sampling approach from parents attending the Department of Pediatrics and the Department of Pediatric and Preventive Dentistry for their first visit. Although restricting recruitment to first-time visitors reduced the likelihood that participants had previously received oral health education at the study institution, the healthcare-based recruitment setting may still have introduced selection bias, as these parents were already seeking healthcare services for their children and may have possessed greater health awareness and healthcare-seeking behavior than the general parent population. Consequently, the generalizability of the findings may be limited. Furthermore, unequal representation of socioeconomic groups within the sample may have influenced the observed associations. The primary education subgroup contained only six participants (1.6%), which may have reduced the statistical power and stability of estimates for comparisons involving this category.

The study relied on self-reported data collected through an electronic questionnaire and did not include clinical verification of the children's oral health status. Consequently, the findings may be subject to recall bias and social desirability bias. In addition, the cross-sectional design precludes the establishment of causal relationships between socioeconomic factors and parental awareness or attitudes. The multivariable models simultaneously adjusted for education and income because these were the primary socioeconomic variables of interest and the principal exposures under investigation. The objective of the adjusted analyses was to estimate the independent association of each socioeconomic indicator while controlling for the other. Additional covariates were not included in the regression models because the study was not specifically designed to evaluate a broader set of predictors, and inclusion of additional covariates was beyond the primary analytical objectives of the study and may not have materially altered interpretation of the socioeconomic associations under investigation.

### Implications

Community based parental education programmes, anticipatory guidance during pediatric healthcare visits, school-based oral health promotion initiatives, and targeted counselling regarding fluoride use and timely first dental visits may help address the identified knowledge gaps. Future public health strategies should focus on integrating oral health education into antenatal care, pediatric healthcare services, school health programmes, and community outreach initiatives to reduce socioeconomic disparities, promote early preventive dental practices, and improve children's oral health outcomes.

## Conclusion

The present study demonstrated that, although parental awareness of basic oral hygiene practices was relatively high, important knowledge gaps persisted regarding fluoride use, preventive oral health measures, and the importance of maintaining deciduous dentition. Multivariable logistic regression with simultaneous adjustment for education and income showed that higher educational attainment was the strongest independent predictor of fluoride knowledge (aOR = 3.46) and regular dental checkup behavior (aOR = 2.32), while higher income independently predicted awareness of primary tooth effects on permanent dentition (aOR = 2.21) and equal care for primary teeth (aOR = 2.04). The tendency to delay the first dental visit until onset of symptoms was observed across all socioeconomic groups regardless of education or income level, highlighting the need for population-wide oral health education and preventive interventions aimed at improving early dental care-seeking behaviour.

## Data Availability

The original contributions presented in the study are included in the article/Supplementary Material, further inquiries can be directed to the corresponding author.
