# Supplementary material for: Awareness and attitudes of parents towards pediatric dentistry and children's oral health: a cross-sectional study
Source: Front Oral Health. 2026 Jun 30;7:1876757. doi: 10.3389/froh.2026.1876757 (PMC13367345; doi:10.3389/froh.2026.1876757)
Supplement: Supplementary file 1 [file Datasheet1.pdf]

Supplementary Figure 1. Participant Flow Diagram

Parents approached for eligibility assessment (n = 389)

▼

Declined to participate (n = 5)

▼

Eligible participants who provided informed

Consent and were enrolled (n = 384)

▼

Completed questionnaire survey

(n = 384)

▼

Responses assessed for completeness

(n = 384)

▼

Included in final statistical analysis

(n = 384)

Figure 1: Flow diagram illustrating participant recruitment, eligibility assessment, questionnaire completion, and inclusion in the final analysis. Of the 389 parents approached, 5 declined participation. A total of 384 parents provided informed consent, completed the questionnaire, and were included in the final analysis.

STROBE CHECKLIST

Item No.	Recommendation	Reported on Page No.
**Title and Abstract**		
1(a)	Indicate the study's design with a commonly used term in the title or abstract	Abstract, Title (Page 1)
1(b)	Provide an informative and balanced summary of what was done and what was found	Abstract (Page 1)
**Introduction**		
2	Explain the scientific background and rationale for the investigation	Introduction (Page 1-2)
3	State specific objectives and any prespecified hypotheses	Introduction (Page 2)
**Methods**		
4	Present key elements of study design early in the paper	Methods (Page 2)
5	Describe the setting, locations, and relevant dates, including periods of recruitment and data collection	Methods (Page 2)
6(a)	Give eligibility criteria and sources/methods of participant selection	Methods (Page 3)
7	Clearly define all outcomes, exposures, predictors, potential confounders, and effect modifiers	Methods (Page 3)
8	For each variable of interest, give sources of data and details of methods of assessment	Methods (Page 2-3)
9	Describe efforts to address potential sources of bias	Methods and Limitations (Page 3 and 13)
10	Explain how study size was determined	Sample Size Calculation (Page 3)

Item No.	Recommendation	Reported on Page No.
11	Explain how quantitative variables were handled in the analyses	Statistical Analysis (Page 3-4)
12(a)	Describe all statistical methods, including those used to control for confounding	Statistical Analysis (Page 3-4)
12(b)	Describe any methods used to examine subgroups and interactions	Statistical Analysis (Page 3-4)
12(c)	Explain how missing data were addressed	No missing data were observed
12(d)	If applicable, describe analytical methods accounting for sampling strategy	Not applicable
12(e)	Describe any sensitivity analyses	Not applicable
**Results**		
13(a)	Report numbers of individuals at each stage of the study	Results page 4; Supplementary material Participant Flow Diagram (Page 3)
13(b)	Give reasons for non-participation at each stage	Supplementary material Participant Flow Diagram (Page 3)
13(c)	Consider use of a flow diagram	Supplementary material Participant Flow Diagram (Page 3)
14(a)	Give characteristics of study participants	Results page 4; Table 1 (Page 5-7)
14(b)	Indicate number of participants with missing data for each variable of interest	No missing data were observed for any variable.
15	Report numbers of outcome events or summary measures	Results (Pages 4-5); Tables 1–4 (Pages 5-10)
16(a)	Give unadjusted estimates and, if applicable, adjusted estimates with precision (e.g., 95% CI)	Table 4 (Page 10)
16(b)	Report category boundaries when continuous variables were categorized	Table 1(Page 5-7)
16(c)	If relevant, translate estimates of relative risk into absolute risk	Not applicable
17	Report other analyses done (subgroups, interactions, logistic regression)	Table 4 (Page 10)

Item No.	Recommendation	Reported on Page No.
**Discussion**		
18	Summarize key results with reference to study objectives	Discussion (Page 12-13)
19	Discuss limitations of the study, considering potential sources of bias or imprecision	Discussion/Limitations (Page 13)
20	Give a cautious overall interpretation of results considering objectives, limitations, multiplicity of analyses, and evidence from similar studies	Discussion (Page 12-13)
21	Discuss the generalizability (external validity) of the study results	Discussion/Limitations (Page 13)
**Other Information**		
22	Give the source of funding and the role of funders	Funding Statement (Page 14)
